# Through-the-scope twin clip for endoscopic closure of gastrointestinal defects: efficacy, safety, and factors influencing closure speed

**DOI:** 10.1007/s00464-025-12504-8

**Published:** 2025-12-29

**Authors:** Chenyang Li, Chao Chen, Fuxiu Huang, Ningning Zhang, Tao Wang, Yan Xu, Zhongrui Xu, Hui Zhao, Zhimin Liang, Shuling Li, Yujie Feng

**Affiliations:** 1 Department of Gastroenterology, The First Medical Center of PLA General Hospital, Beijing, China; 2 Department of Gastroenterology, The Fourth Medical Center of PLA General Hospital, Beijing, China

**Keywords:** Through-the-scope twin clip, Endoscopic closure, Defect closure, Super minimally invasive surgery

## Abstract

**Background and aims:**

This study aimed to evaluate the efficacy, safety, and factors influencing closure speed of the through-the-scope twin clip (TTS-TC) for endoscopic defect closure.

**Methods:**

A retrospective observational study was conducted at a single center, involving patients who underwent endoscopic closure using the TTS-TC from December 2022 to December 2024.

**Results:**

TTS-TC was successfully deployed in all 78 patients, yielding a complete closure rate of 98.7% (78/79 defects). The median defect area was 10.5 (6.3–15.8) cm^2^. Median procedure time was 15.0 (10.0–19.0) min, corresponding to a median closure speed of 0.73(0.45, 1.07) cm^2^/min. Univariate and multivariate analyses identified operator experience and defect area as independent factors of closure speed. Early complications included local peritonitis in 3.8% (3/78) and infection in 1.3% (1/78); delayed bleeding and long-term adverse events did not occur.

**Conclusions:**

The TTS-TC emerges as a promising tool for the closure of diverse gastrointestinal defects, offering a new dimension in endoscopic management.

**Graphical abstract:**

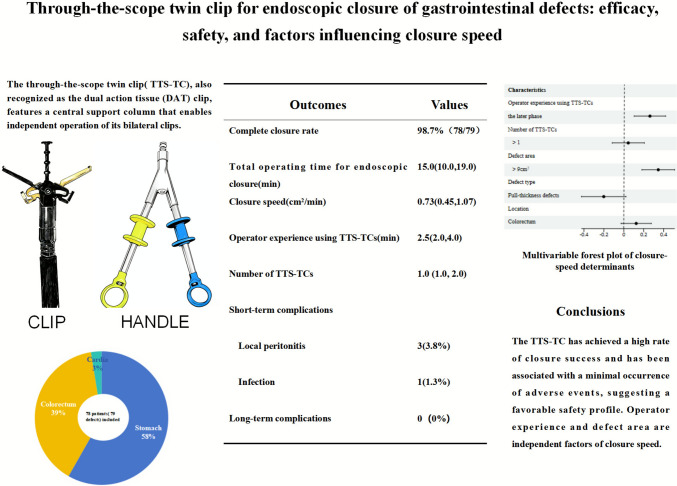

**Supplementary Information:**

The online version contains supplementary material available at 10.1007/s00464-025-12504-8.

Over the past two decades, endoscopic super minimally invasive surgery (SMIS) has matured from a pioneering concept into of the standard care for early gastrointestinal neoplasia and subepithelial tumors. By preserving organ integrity while eradicating disease, SMIS has delivered substantial benefits to patients. Achieving “super-safety,” however, is equally imperative, and secure endoscopic closure of the post-resection defect is pivotal. Delayed bleeding complicates 5–10% of gastric SMIS procedures, and delayed perforation occurs in < 3%; after colonic SMIS, these rates are 2.7% and 5.2%, respectively [1]. Prophylactic defect closure significantly reduces these complications [2–6], underscoring its critical role in minimizing adverse events and ensuring “super-safety” after SMIS.

Traditional through-the-scope clips (TTSCs) remain the most widely used devices, yet their limited jaw span impedes reliable apposition of the mucosal margins of large defects, confining their utility to wounds < 2 cm [7, 8]. Closure of large (> 2 cm) or full-thickness gastrointestinal defects therefore continues to pose a formidable challenge in SMIS.

The recently introduced through-the-scope twin clip (TTS-TC), featuring a bilateral clip design, has demonstrated distinct advantages in large-defect closure and may address this unmet need [9]. Nonetheless, robust cohort data assessing the efficacy and safety of TTS-TC across lesions of varying location, size, depth, and etiology are lacking. We therefore conducted this retrospective analysis of real-world outcomes to provide evidence and practical guidance for broader adoption of TTS-TC.

## Methods

### Setting and participants

From December 2022 to December 2024, 78 patients (79 defects) underwent TTS-TC placement consecutively due to ulcer bleeding, deep wall defects, or perforations in the stomach, colon, or rectum. Patients without TTS-TC closure or lost to follow-up were excluded. Informed consent was secured from all participants prior to undergoing endoscopic interventions, in accordance with ethical standards. All procedures were performed by two highly experienced endoscopists. The procedures were performed on conscious patients or those under deep sedation with the support of an anesthesiologist. This study was approved by the Biomedical Research Ethics Committee of the Fourth Medical Center of Chinese PLA General Hospital.

### The TTS-TC system

**Fig. 1:**
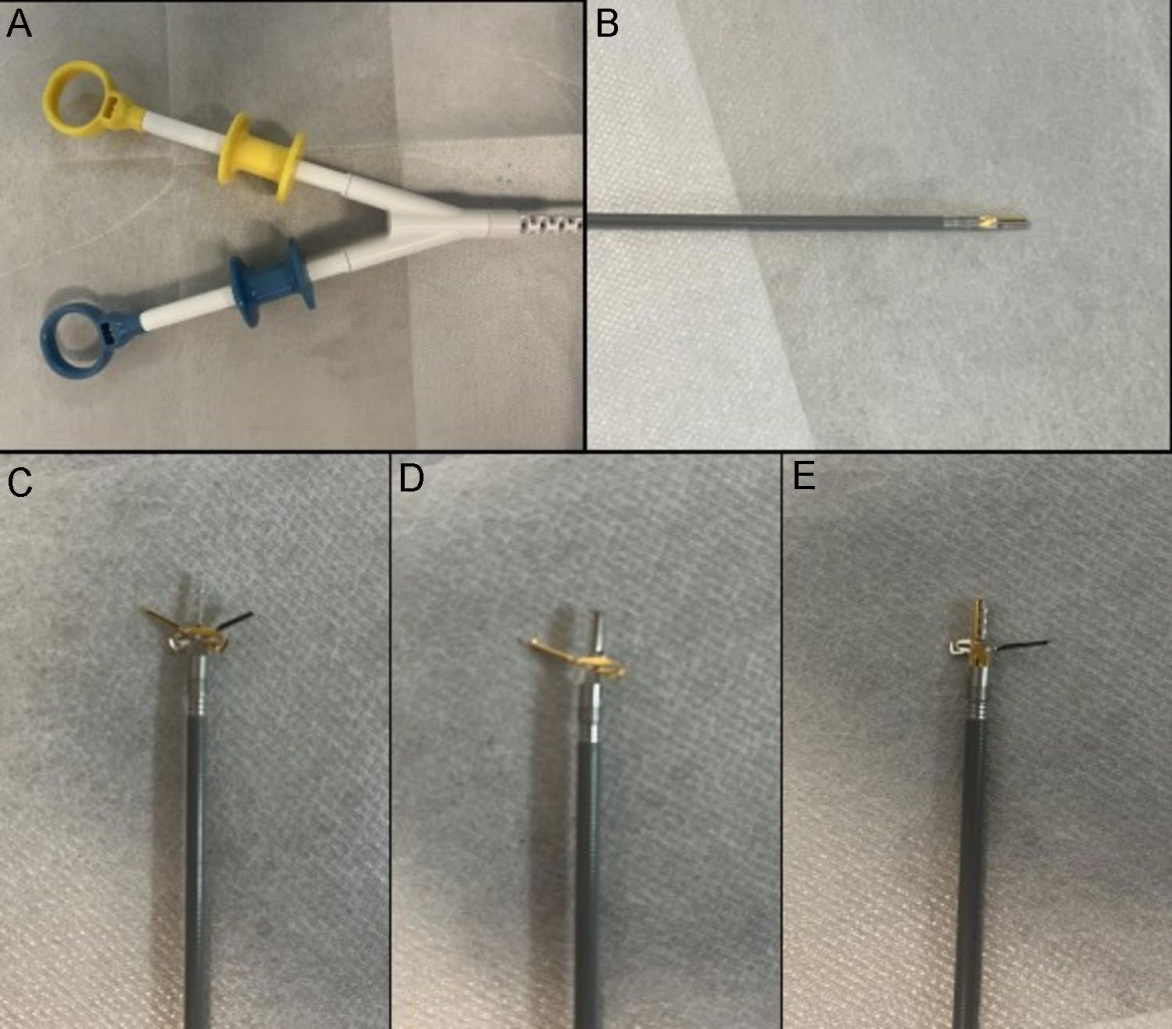
The through-the-scope twin clip. The clips are controlled independently by two handles. The gold clip is controlled by the gold handle, and the blue clip is controlled by the blue handle. **A** Operation Handles. **B** Both clips closed. **C** Both clips opened. **D** Gold clip opened only. **E** Blue clip opened only

The main endoscopic instruments in this study were a gastroscope (Q260J; Olympus, Tokyo, Japan), IT knife(Olympus), conventional TTSC (ROCC-D-26-195-C and ROCC-F-26-195-C; Micro-Tech Co, Ltd, Nanjing, China), and the novel TTS-TC (Micro-Tech Co, Ltd, Nanjing, China). Compared to TTSC, a distinctive feature of the TTS-TC is the presence of an additional fixed support column, centrally positioned between the metal clips on both sides, which facilitates independent operation (Fig. 1). The bilateral clips operate independently by two color-coded handles, with a maximum opening angle of 60 degrees.

### Operation procedure

**Fig. 2:**
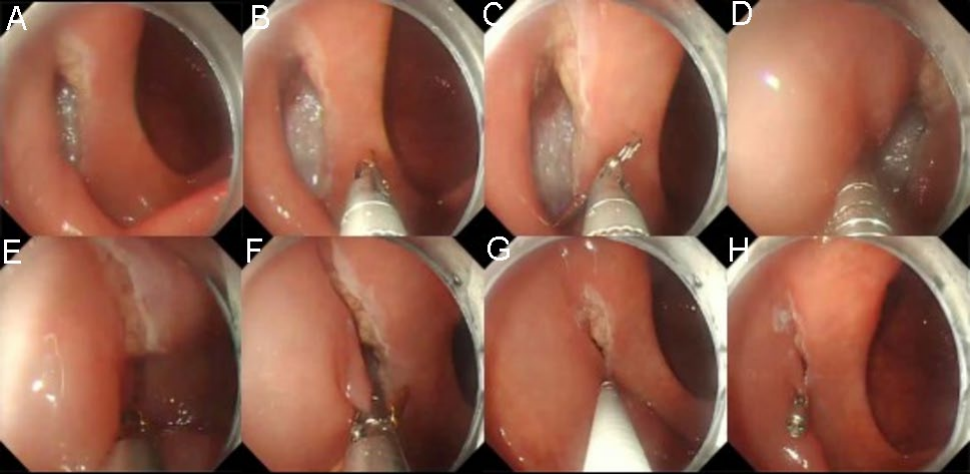
Operation steps using TTS-TC for wound closure. **A** A wound was located in the ascending colon. **B** The TTS-TC was inserted into the colon cavity through the endoscopic working channel. **C** The clip on one side of the TTS-TC was opened. **D** The clip clamped the mucosa on one side of the wound. **E** The clamped mucosa was pulled toward the opposite of the wound. **F** The clip on the other side of the TTS-TC was opened. **G** The clip clamped the mucosa on the other side of the wound. **H** The clips on both sides of the TTS-TC were locked and released, resulting in the large wound becoming several small wounds

The TTS-TC was introduced into the gastrointestinal tract via the endoscope’s working channel (Fig. 2, Video 1). One side of the TTS-TC was opened to clamp the edge of the defect. The mucosa, once clamped, was carefully repositioned to approximate the opposite edge of the defect. The clip on the other side of the TTS-TC was then opened to clamp the opposite edge. Once both edges of the defect were secured, the clips were locked. The TTS-TC was then released and retained in vivo. Additional clips were deployed to achieve complete closure.

### Data collection

Demographic information about patients, lesion characteristics, endoscopic closure time, procedural outcomes, and post-procedural outcomes, including adverse events, were extracted from electronic medical records. Follow-up assessments were conducted at the discretion of the endoscopist, based on the perceived necessity. Telephone follow-up was conducted on post-operative days 7 and 28.

### Outcomes

Complete closure was defined as complete apposition of the mucosal defect margins with no visible submucosal exposure > 3 mm along the closure line, regardless of whether additional traditional through-the-scope clips were used. The operating time using TTS-TCs was defined as the duration between clip insertion and release. The total operating time for endoscopic closure was defined as the duration between the end of dissection or hemostasis and complete closure of the defect. Closure speed was calculated as the area of the defect divided by the closure time. Full-thickness resection was defined as the presence of all gastrointestinal wall segments in the resected specimen. Adverse events were categorized as short-term (within 7 days after operation) or long-term (within 28 days after operation) complications, including delayed bleeding, perforation, local peritonitis, and infection.

### Statistical analysis

Descriptive statistics were calculated for all demographic, imaging, and clinical variables and were reported as median (Q1, Q3), or as a proportion. Univariate analysis was performed by using the chi-square test and the Fisher exact test for categorical variables and the t-test for continuous variables as required. Factors influencing closure speed were examined by linear regression. All statistical analyses were conducted by using SPSS version 22.0 (IBM, Armonk, NY). A *p*-value < 0.05 was considered significant.

## Results

TTS-TC was used to achieve definitive closure in 78 patients harboring 79 gastrointestinal defects (Table 1). The cohort comprised 66 post-SMIS non-full-thickness defects, 10 post-SMIS full-thickness defects, 2 bleeding gastric ulcers, and 1 delayed post-SMIS colonic perforation. Anatomically, 46 defects were located in the stomach, 2 at the cardia, and 31 throughout the colorectum. The median defect area measured 10.5(6.3, 15.8) cm^2^; the largest—a 6.0 × 5.0 cm non-full-thickness sigmoid lesion—was securely sealed in approximately 18 min. These findings confirm that TTS-TC can reliably and expeditiously close a wide spectrum of large and anatomically diverse gastrointestinal defects.

**Table 1:** Demographic and clinical characteristics of patients in the study

Characteristics	Values
Age (years)	64 (56,70)
Male gender	48 (61.5%)
Defect type	
Post-SMIS non-full-thickness defects	66 (83.5%)
Post-SMIS full-thickness defects	10 (12.7%)
Bleeding gastric ulcers	2 (2.5%)
Delayed post-SMIS colonic perforation	1 (1.3%)
Location	
Stomach	46
Gastric antrum	15 (32.6%)
Gastric body	15 (32.6%)
Gastric angle	2 (4.4%)
Gastric fundus	14 (30.4%)
Colorectum	31
Rectum	7 (22.6%)
Sigmoid colon	14 (45.2%)
Descending colon	2 (6.5%)
Transverse colon	2 (6.5%)
Ascending colon	4 (12.9%)
Cecum	2 (6.5%)
Cardia	2
Defect area (cm^2^)	10.5 (6.3,15.8)
Stomach	9.2 (5.5,12.3)
Colorectum	14.0 (9.6,20.0)
Cardia	6.25

*SMIS* super minimally invasive surgery

### Study outcome

**TTS-TC closure.** TTS-TC was attempted in 79 gastrointestinal defects, achieving complete closure in 98.7% (78/79) (Table 2). The sole failure was a 4.0 × 4.0 cm, bleeding gastric-angle ulcer; the device successfully sealed approximately 5/6 of the lesion (Supplementary Fig S1). Median operating time using TTS-TCs was 2.5(2.0, 4.0) min. During the initial 20 cases, it averaged 3.0(3.0, 5.0) min, falling to 2.0(2.0, 3.0) min in the subsequent 59 cases—a 37.8% reduction (*p* < 0.05). On average, 1.0 (1.0, 2.0) clips were used per defect: 1.0 (1.0, 2.0) for gastric, 1.0 for cardial, and 1.0 (1.0, 2.0) for colorectal defects. We further investigated the potential factors affecting the usage of TTS-TCs and TTSCs in Supplementary Table S1 and Supplement Table S2. The results indicated that larger defect areas were associated with a higher number of TTS-TCs used (*p* < 0.001). Operator experience in using TTS-TCs influenced the number of TTSCs used (*p* = 0.02), while defect area and the number of TTS-TCs had no significant impact on the number of TTSCs used (*p* > 0.05). Overall mean endoscopic closure time was 14.0(10.0, 20.0) min in the stomach, 13.5(12.0, 15.0)min at the cardia, and 15.0(11.0,18.0)min in the colorectum.

**Table 2:** Clinical outcomes of defect closure and operative procedures

Outcomes	Values	P value
Complete closure rate	98.7% (78/79)	> 0.999
Stomach	97.8% (45/46)	
Cardia	100% (2/2)	
Colorectum	100% (31/31)	
Total operating time for endoscopic closure (min)	15.0 (10.0,19.0)	0.943
Stomach	14.0 (10.0,20.0)	
Cardia	13.5 (12.0,15.0)	
Colorectum	15.0 (11.0,18.0)	
Closure speed (cm^2^/min)	0.73 (0.45,1.07)	**0.002**
Stomach	0.54 (0.39,0.82)	
Cardia	0.47 (0.42,0.52)	
Colorectum	0.92 (0.74,1.20)	
Operator experience using TTS-TCs (min)	2.5 (2.0,4.0)	**0.001**
the initial learning phase (first 20 cases)	3.0 (3.0, 5.0)	
the later phase (subsequent 59 cases)	2.0 (2.0, 3.0)	
Number of TTS-TCs	1.0 (1.0, 2.0)	0.593
Stomach	1.0 (1.0, 2.0)	
Cardia	1.0 (1.0, 1.0)	
Colorectum	1.0 (1.0, 2.0)	

The bolded data indicates that the differences are statistically significant

**Fig. 3:**
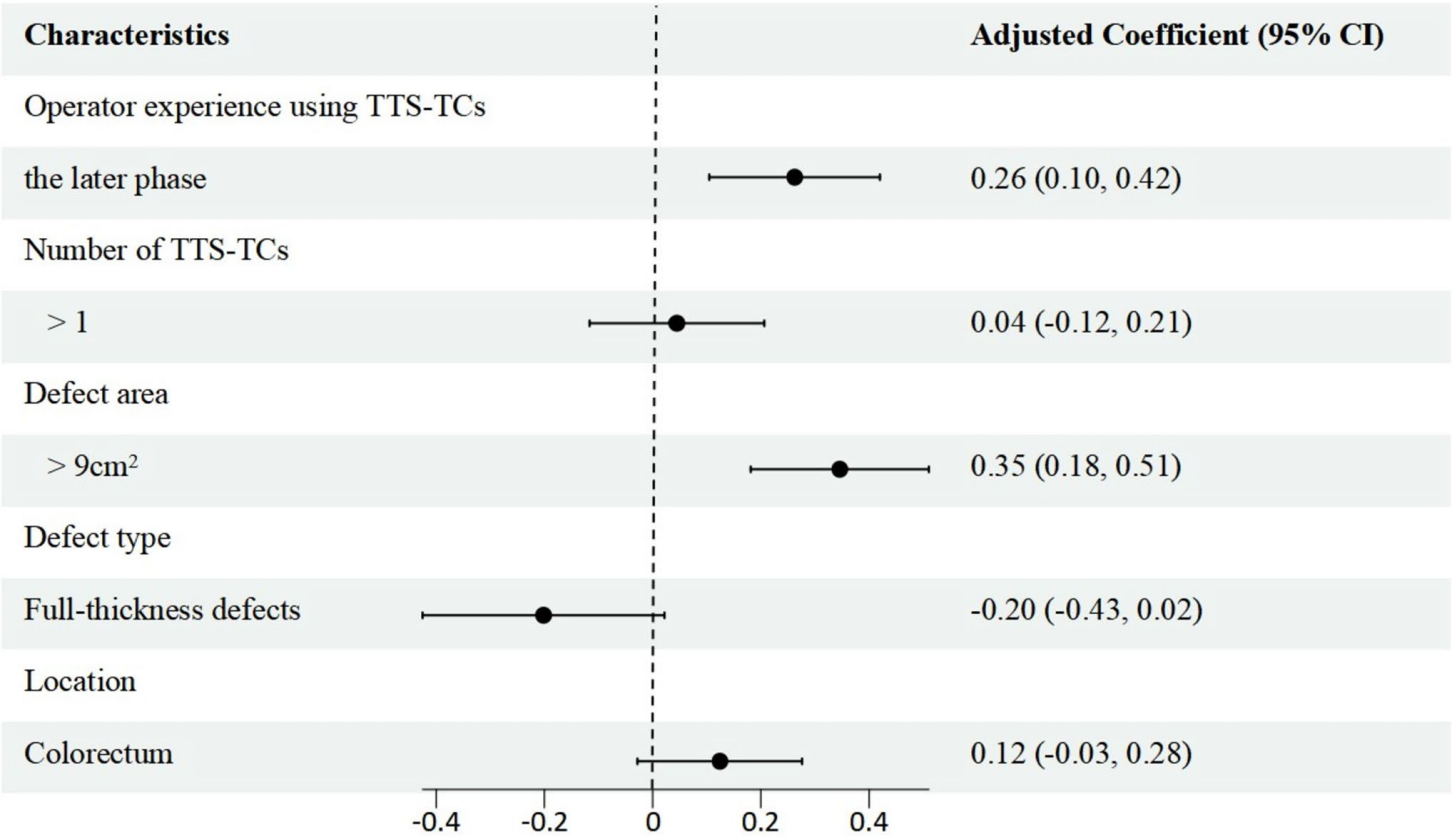
Multivariable forest plot of closure-speed determinants

Median closure speed was 0.73(0.45, 1.07) cm^2^/min and varied significantly by location: slowest at the cardia (0.47 cm^2^/min), intermediate in the stomach (0.54 cm^2^/min), and fastest in the colon (0.92 cm^2^/min). Speed improved substantially after the learning curve (early phase 0.49 vs late phase 0.80 cm^2^/min; *p* < 0.05). Defects closed with > 1 clip achieved a higher speed (0.81 cm^2^/min) than those requiring only one clip (0.59 cm^2^/min; *p* < 0.05). Non-full-thickness defects closed more rapidly than full-thickness defects (0.78 vs 0.35 cm^2^/min; *p* < 0.05), and large defects (> 9 cm^2^) were sealed faster than smaller ones (≤ 9 cm^2^; 0.92 vs 0.46 cm^2^/min; *p* < 0.05). Multivariable linear regression identified operator experience and defect area as the only independent predictors of closure speed (Table 3, Fig. 3).

**Table 3:** Univariate analyses of factors influencing the closure speed of defects in TTS-TC closure

Characteristics	Closure speed (cm^2^/min)	Beta (95%CI)	*P* value
Operator experience using TTS-TCs			
the initial learning phase	0.49 (0.41, 0.65)	0.29 (0.09, 0.48)	**0.005**
the later phase	0.80 (0.50, 1.11)		
Time of initial TTS-TC deployment (min)			
≤ 2	0.80 (0.42, 1.11)	−0.06 (-0.24, 0.12)	0.494
˃ 2	0.64 (0.45, 0.96)		
Number of TTS-TCs			
≤ 1	0.59 (0.41, 0.90)	0.24 (0.06, 0.42)	**0.010**
˃ 1	0.81 (0.58, 1.23)		
Location			
Stomach and Cardia	0.53 (0.40, 0.81)	0.28 (0.11, 0.45)	**0.002**
Colorectum	0.92 (0.74, 1.20)		
Defect area (cm^2^)			
≤ 9	0.46 (0.33, 0.60)	0.46 (0.30, 0.61)	** < 0.001**
˃ 9	0.92 (0.66, 1.20)		
Defect type			
Non-full-thickness defects	0.78 (0.50, 1.09)	−0.41 (-0.66, -0.16)	**0.002**
Full-thickness defects	0.35 (0.28, 0.50)		

The bolded data indicates that the differences are statistically significant

**Adverse events.** Among 78 patients undergoing endoscopic defect closure with the TTS-TC, two developed fever (38.1–38.4 °C) and abdominal pain on post-operative day 7. Examination showed localized tenderness without guarding or rebound, imaging ruled out perforation, and both episodes of focal peritonitis resolved within 1–3 days. One patient with a full-thickness defect experienced severe abdominal pain and diffuse tenderness with mild rebound but remained afebrile; no perforation was demonstrated, the episode was attributed to focal peritonitis, and symptoms abated by day 3. A fourth patient had a transient temperature rise to 37.8℃ without pain or other symptoms, consistent with mild local infection, and returned to baseline within 24 h. Telephone follow-up at 7 and 28 days revealed no delayed bleeding or perforation in any patient. Thus, early local peritonitis occurred in 3.8% (3/78), infection in 1.3% (1/78), and no long-term complications were observed (Table 4).

**Table 4:** Adverse events

Characteristics	Values
Short-term complications	
Delayed bleeding	0 (0%)
Perforation	0 (0%)
Local peritonitis	3 (3.8%)
Infection	1 (1.3%)
Long-term complications	
Delayed bleeding	0 (0%)
Perforation	0 (0%)
Local peritonitis	0 (0%)
Infection	0 (0%)

## Discussion

Reliable endoscopic closure of gastrointestinal defects—particularly large or full-thickness wounds—substantially lowers the risk of bleeding, perforation, and infection after super minimally invasive surgery, making it a cornerstone of procedural safety. Available techniques range from traditional through-the-scope clips (TTSC) and over-the-scope clips (OTSC) to purse-string devices that combine clips with loops or sutures, dedicated endoscopic suturing systems (OverStitch, X-Tack), and fibrin sealant application[10–16]. Each approach carries its own merits and drawbacks. Delivering a rapid, effective, and economical closure for defects exceeding 2 cm remains a central challenge in endoscopic super minimally invasive surgery.

The TTS-TC, also recognized as the dual action tissue (DAT) clip, features a central support column that enables independent operation of its bilateral clips, with a maximum outer diameter of 2.9 mm, an opening angle of up to 60 degrees, and an opening size of 1.0 cm, making it suitable for effective closure of wounds less than 5.0 cm in diameter. Like other TTSCs, the TTS-TC is simple and flexible: it passes directly through the working channel, eliminating OTSC’s need to withdraw and reload the endoscope. The clip can be repeatedly opened, closed, and rotated in situ for position adjustments, markedly lowering the technical burden and shortening procedure time for large defects. We were the first to report its clinical use—securely closing a 4.0 × 3.0 cm post-ESD defect after gastric stromal tumor resection and a semicircumferential 5.0 × 4.0 cm post-ESD defect following endoscopic resection of a lateral-spreading rectal tumor [17, 18]. Subsequent single cases and small colonic series have appeared [19, 20], yet large-scale evidence across multiple GI sites and defect types remains absent.

We retrospectively evaluated the TTS-TC for defect closure at every major GI site—cardia; stomach (antrum, body, fundus, angularis); and the entire colorectum (rectum, sigmoid, descending, transverse, ascending, cecum). Complete closure was achieved in 98.7% (78/79) of cases; the single failure was a 4.0 × 4.0 cm bleeding ulcer at the gastric angularis. Extensive fibrosis, the difficult angulation, and early-operator inexperience limited closure to 5/6 of the defect; the remaining posterior wall was left open. The patient nevertheless healed without bleeding. These data confirm that the TTS-TC can reliably seal defects across the GI tract—including anatomic niches traditionally regarded as challenging—and accommodate diverse defect types (non-full-thickness defects, full-thickness defects, bleeding ulcers, delayed perforations). Prospective trials may now explore its utility in even more complex settings such as fistulas or sinuses.

This study confirms that the TTS-TC achieves a high complete closure rate (98.7%) and outperforms prior reports in both closure time and speed, underscoring its ease of mastery and ability to seal defects rapidly and reliably. Closure speed varied by site: slowest at the cardia, intermediate in the stomach, and fastest in the colon. The sluggish cardia rate reflects limited workspace and awkward angles, whereas the stiffer gastric mucosa demands greater traction than the pliable colonic wall. The small cardia cohort, however, necessitates larger series before definitive conclusions. Operator experience exerted a pronounced effect: closure speed rose 37.8% from the early to the late phase, indicating that even a brief learning curve meaningfully shortens procedure time and maximizes efficiency. Closure speed also correlated with clip number. Early deployment of multiple TTS-TCs—especially for large or difficult lesions—quickly approximates defect edges and accelerates overall closure. Yet additional clips inflate costs; future work should therefore delineate an optimal defect area-site-clip number algorithm that preserves efficacy without superfluous expenditure. Our analysis of the potential factors affecting the usage of TTS-TCs and TTSCs revealed that larger defect areas are associated with a higher number of TTS-TCs used, which aligns with our practical experience. Moreover, the number of TTSCs used increases with the operator’s experience in using TTS-TCs. We speculate that this is because experienced operators are more likely to subdivide larger defects into multiple smaller ones, thereby increasing the number of TTSCs used. Additionally, the process of using TTS-TCs to break down larger defects into smaller ones creates a complex interrelationship among defect area, the number of TTS-TCs, and the number of TTSCs used. This relationship warrants further investigation to be fully understood. Full-thickness defects closed more slowly than non-full-thickness lesions, likely owing to greater technical complexity, impaired luminal visualization, and stricter demands for watertight apposition. Paradoxically, larger defects closed faster. We hypothesize that once TTS-TCs achieve initial approximation, subsequent ordinary clips are applied with ease, yielding an exponential closure curve. This benefit, however, may not extend to exceptionally large defects that resist initial gathering.

Extensive hands-on experience has yielded three practical TTS-TC tips. First, position before you close: open the clip arms parallel to the wound edges, then advance and rotate slowly—patiently synchronized with your assistant—until orientation is perfect. Second, give the clip room to work: extend it 1–2 cm beyond the scope tip so the arms deploy fully, and decide in advance which mucosal lip to grasp first so the opposite side stays in clear view. Third, map large wounds before you start: for defects ≥ 4–5 cm, sketch the closure line, anticipate each clip site, and limit every bite (2–3 cm in cardia or stomach, 3–4 cm in most colonic segments, up to 5 cm only in capacious colon) to prevent arm fracture from excessive traction. By choreographing the path, shortening each step, and adding clips judiciously, even giant defects can be closed completely and safely.

The study is limited by its retrospective, single-center design and attendant selection bias. The lack of head-to-head data against traditional clips, OTSC, or other closure techniques prevents definitive conclusions. Multicenter, prospective, randomized trials are therefore essential to confirm these findings, guide optimal defect-closure choices, and refine clinical adoption of the TTS-TC.

## Conclusions

In summary, the TTS-TC has demonstrated robust reliability and efficacy as an endoscopic tool for managing an array of gastrointestinal defects. Its application has been effective in addressing issues arising from bleeding, perforations, and defects after endoscopic resections. The TTS-TC has achieved a high rate of closure success and has been associated with a minimal occurrence of adverse events, suggesting a favorable safety profile. Operator experience and defect area are independent factors of closure speed. Nevertheless, further research is encouraged to validate these preliminary findings and explore the broader application of TTS-TC in diverse clinical scenarios.

## Supplementary Information

Below is the link to the electronic supplementary material.

Supplementary file1 (DOCX 29 KB)

Supplementary file2 (DOCX 28 KB)

Supplementary file3 (PNG 2382 KB)

Supplementary file4 (MP4 238800 KB)

## Data Availability

The data that supports the findings of this study are available in the supplementary material of this article.
