# Supplementary material for: Through-the-scope twin clip for endoscopic closure of gastrointestinal defects: efficacy, safety, and factors influencing closure speed
Source: Surg Endosc. 2025 Dec 29;40(2):1711–8. doi: 10.1007/s00464-025-12504-8 (PMC12881058; doi:10.1007/s00464-025-12504-8)
Supplement: Supplementary file 1 — Supplementary file1 (DOCX 29 KB) [file 464_2025_12504_MOESM1_ESM.docx]

**Supplement Table 2** Univariable and multivariable analysisof factors influencing the number of TTSCs used in closure.

| Characteristics（n） | Number of TTSCs | | P value | |
| --- | --- | --- | --- | --- |
|  | ＜10 | ≥10 | Univariable analysis | Multivariable analysis |
| Operator experience using TTS-TCs |  |  | **0.020** | **0.020** |
| the initial learning phase | 14 | 6 |  |  |
| the later phase | 23 | 26 |  |  |
| Location |  |  | 0.811 |  |
| Stomach and Cardia | 23 | 15 |  |  |
| Colorectum | 14 | 17 |  |  |
| Defect area (cm^2^) |  |  | 0.995 |  |
| ≤ 9 | 15 | 17 |  |  |
| ˃ 9 | 22 | 25 |  |  |
| Number of TTS-TCs |  |  | 0.866 |  |
| ≤ 1 | 13 | 14 |  |  |
| ˃ 1 | 24 | 28 |  |  |
| Defect type |  |  | 0.263 |  |
| Non-full-thickness defects | 34 | 35 |  |  |
| Full-thickness defects | 3 | 7 |  |  |
