# Supplementary material for: Through-the-scope twin clip for endoscopic closure of gastrointestinal defects: efficacy, safety, and factors influencing closure speed
Source: Surg Endosc. 2025 Dec 29;40(2):1711–8. doi: 10.1007/s00464-025-12504-8 (PMC12881058; doi:10.1007/s00464-025-12504-8)
Supplement: Supplementary file 2 — Supplementary file2 (DOCX 28 KB) [file 464_2025_12504_MOESM2_ESM.docx]

**Supplement Table 1** Univariable and multivariable analysisof factors influencing the number of TTS-TCs used in closure.

| Characteristics（n） | Number of TTS-TCs | | P value | |
| --- | --- | --- | --- | --- |
|  | ≤ 1 | ˃ 1 | Univariable analysis | Multivariable analysis |
| Operator experience using TTS-TCs |  |  | 0.649 |  |
| the initial learning phase | 14 | 6 |  |  |
| the later phase | 38 | 21 |  |  |
| Location |  |  | 0.844 |  |
| Stomach and Cardia | 32 | 16 |  |  |
| Colorectum | 20 | 11 |  |  |
| Defect area (cm^2^) |  |  | **<0.001** | **<0.001** |
| ≤ 9 | 29 | 3 |  |  |
| ˃ 9 | 23 | 24 |  |  |
| Defect type |  |  | 0.118 |  |
| Non-full-thickness defects | 43 | 26 |  |  |
| Full-thickness defects | 9 | 1 |  |  |
