# Supplementary figures and images for: Through-the-scope twin clip for endoscopic closure of gastrointestinal defects: efficacy, safety, and factors influencing closure speed
Source: Surg Endosc. 2025 Dec 29;40(2):1711–8. doi: 10.1007/s00464-025-12504-8 (PMC12881058; doi:10.1007/s00464-025-12504-8)

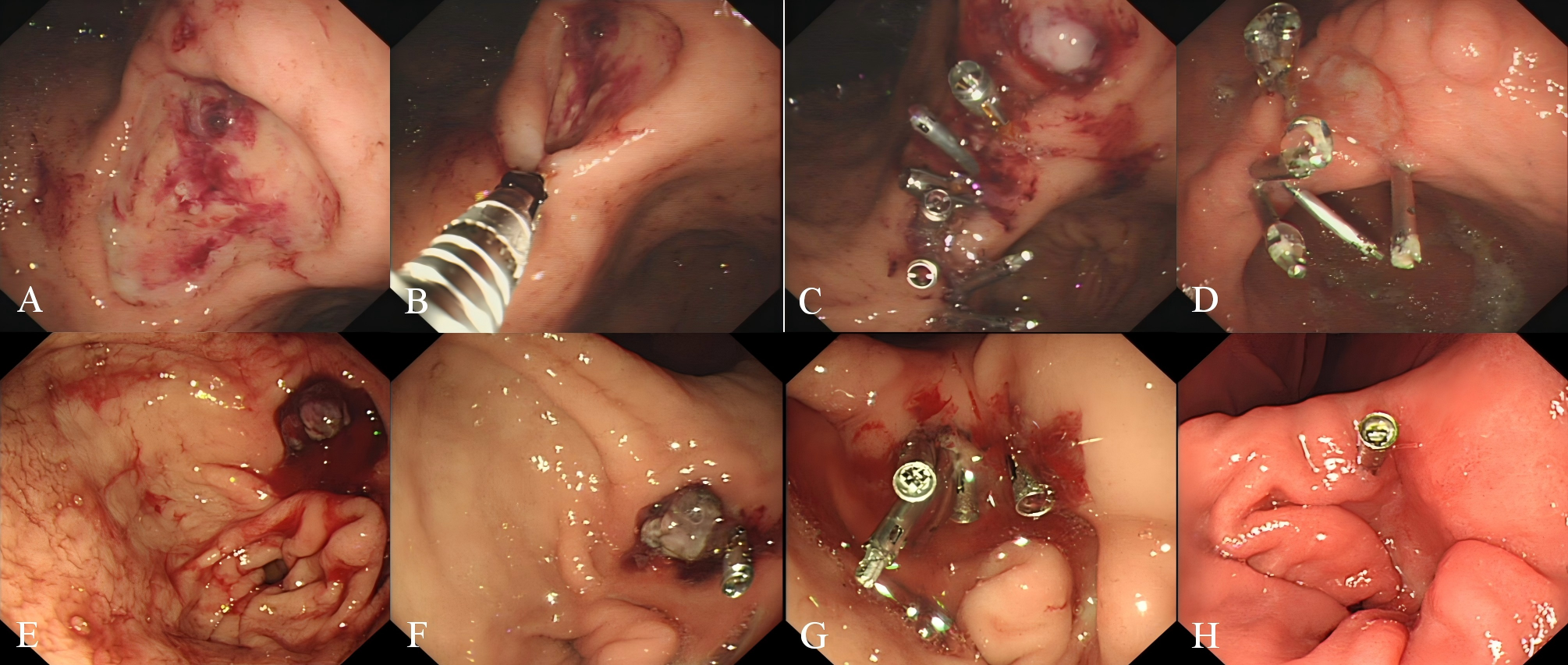

Supplement: Supplementary file 3 — Supplementary file3 (PNG 2382 KB) [file 464_2025_12504_MOESM3_ESM.png]
